# Treatment of Pulpless Teeth

**Published:** 1897-10

**Authors:** A. J. Husband

**Affiliations:** Toronto.


ARTICLE IV.

### TREATMENT OF PULPLESS TEETH.

BY A. J. HUSBAND, L. D. S., TORONTO.

**Read before Toronto Dental Society, May 12th, 1897.**

For convenience of description, we will divide pulpless teeth into those presenting with pulps recently dentalized by medication; second, with putrescent pulps; third, with acute alveolar abscess without fistula; fourth, with fistulous opening; and fifth, preparatory to inserting pin crowns.

In the first case we will suppose the pulp devitalized but not removed. Adjust the rubber dam after cutting away the decalcified dentine, make a free opening into the pulp chamber, not hesitating to sacrifice good tooth substance in order to secure light and thoroughness of manipulation. Endeavor to remove the pulp by inserting a barbed brooch well up the root, twisting and withdrawing it with very often the pulp attached. In refractory cases I find that with the Evans' root-drier inserted hot the pulp will adhere to it and come away easily. Having the pulp removed, check the hæmorrhage, if any, by injecting into the canals pyrozone; for this purpose use the minim syringe. Whether there is any bleeding or not, I wash out with pyrozone, followed by sodium-peroxide, this last for the purpose of saponifying any remains which may be left; wash this out with applications of water and dry thoroughly with Evans' drier or hot air or both. Wipe out the roots with oil of cinnamon on a fine brooch, fill with chlora-percha cones to displace surplus liquid. In this class of teeth I deem immediate filling the best.

Second: Putrescent pulps. Secure all the cleanliness possible by removing softened dentine and washing out the cavity well with tepid water before adjusting the dam. After the dam is applied swab out the cavity with pyrozone and evaporate moisture with hot air, secure free opening into canals, reaming them out if the margins are soft; wash out thoroughly with repeated applications of pyrozone. In washing the canals with $H_2O$ great care must be exercised not to use too much at a time. I use the minim syringe and inject a very small drop at a time, wiping out and applying repeatedly until effervesence ceases, followed by sodium-peroxide; wipe out canals with cotton wound round a fine brooch until all discoloration ceases. Again use hot air or Evan's drier, followed by an application of oil of cinnamon, and fill as in the previous case.

If it is impossible to check the discharge I fill the root with cotton saturated with pyrozone and mercury bichloride 1 in 1,000 equal parts and renew every 2 or 3 days until discharges cease.

Third : Acute alveolar abscess without fistula. These cases usually present with swollen faces and extreme soreness of the teeth. My first treatment is simply to secure an opening into the pulp chamber. I dismiss the patient then until soreness disappears, when I can operate comfortably for both of us. I know there is a great outcry against this procedure, but I follow it nevertheless, deeming the treatment equally efficacious and considerably more human than any other that I know. After soreness has disappeared I treat as in former cases.

Fourth : Cases with fistulous opening are treated by opening fully into canals and cleaning them as well as can be with brooches and then forcing pyrozone through the fistula with a hypodermic syringe, using a washer of gutta-percha to dam up the canal to prevent return of liquid. This is followed by aromatic-sulphuric acid injected in the same manner and the roots dried and filled once with chloro-percha and gutta-percha points as formerly. In some cases where it is deemed expedient to open into the canal from the tooth, I follow the track of the fistula with bars, scrape the end of the root, or as I think better still, extract and replant.

Fifth : Before inserting pins for any purpose after treating the root as in the former case I fill the apex with tin to prevent cement from being crowded through.—*Daminion Dental Journal.*

